# Mr. Crichghton on the Efficacy of Vacciola

**Published:** 1804-09-01

**Authors:** J. Cricghton

**Affiliations:** Member of the Royal College of Surgeons in Ireland. Dublin, St. Andrew Street


### *To the Editors of the Medical and Physical Journal.*

GENTLEMEN,

I Hasten to lay before you some Papers just arrived from Dublin. Individually, Members of the Medical Council of the Royal Jennerian Society, I think ye will feel highly gratified, as the contents of them are of more importance than a folio volume of speculative opinions. With respect to the lines relating to myself, I enclose them, in order to show how well founded were my alarms on the spreading abroad of the pernicious pamphlet

*Salisbury Square, 14, viij.* 1804. J. W.

---

To Dr. WALKER.

Resident Inoculator to the Royal Jennerian Society, London.

SIR,

The publication of Mr. Goldson's cases of small-pox subsequent to vaccination, having occasioned some uneasiness in the minds of several persons in this kingdom, I was induced to try its validity by an experiment, the result of which, I hope, will appear so satisfactory to you, (who have shown so much anxiety to promote public confidence in the efficacy of vaccination) that you will have the goodness to make it generally known in every way you may think proper.

I am, &c.

*Dublin, St. Andrew Street, August* 10, 1804.

J. CRICGHTON,
Member of the Royal College of Surgeons in Ireland.

Having had for some years past the medical and surgical control of two great institutions, namely the Foundling Hospital and Dispensary for the Infant Poor and Vaccine

Vaccine Inoculation in Dublin, which have afforded me ample means of co-operating with others in distributing the benefit of vaccine inoculation thoughout this city and kingdom, I feel anxious to contribute, as far as in my power, to restore to every person that confidence in the permanent efficacy of cow-pock inoculation, which truth must ever command, and which a late publication from Mr. Goldson, surgeon, at Portsea, has called in question. As the express object of Mr. Goldson's work is to shew that the vaccine inoculation, although found to be a preventive against the small-pox for two or three years, had, in certain cases, ceased to prove so at the end of that period, the following experiments were made to ascertain this important point.

On the 24th of July last, I made choice of fifteen healthy children, some of whom had been inoculated by me near four years, and all above three years ago, with vaccine infection, (the first used in this kingdom) every one of whom had the disease in the most perfect manner, as appears from the different records kept in the Foundling Hospital, and the distinct mark of vaccine inoculation on the arm of each child; and having procured a child with a great quantity of small-pox infection in its most active state, I committed them to the care of George Stewart, Esq. Surgeon-General, who, with the greatest attention, inserted the variolous matter (taken from the pustules of the small-pock child) into the arms of each of the fifteen children, and afterwards continued them in the same room with the sick child, where they have ever since remained, having constant communication with it in every way, and frequently in the same bed, &c.

On the third day, the punctures in the arms of every child inflamed, and continued to go on in most of them in the usual way of small-pox inoculation until the eighth day, when all gradually began to decline without producing either sickness or eruption; sufficiently evincing that cow-pock inoculation had proved a permanent protection to the constitution of every one of the foregoing children from the small-pox.

Conceiving that a business so highly interesting in its nature could not be conducted with too much precision and publicity, I have great pleasure in subjoining the names of the following respectable professional gentlemen, who were so good as to witness the progress, and who join with with me in congratulating the public on the happy result of these experiments.

JOHN CRUMPTON, Esq. M. D. Member of the College of Physicians.

GEORGE RENNY, Esq. }

RALPH S. O'BREE, Esq. } Members of the Royal College of Surgeons in Ireland.

ABR. COLLES, Esq. }

—— WILSON, Esq. Surgeon, 3d Regiment of Dragoons.

—— JONES, Esq. Assistant Surgeon, ditto.

—— M'Nally, Esq. Surgeon, Armagh Militia.

We are much obliged to Dr. Walker for the valuable communication he has obtained, and think it will, as well as the following, materially tend to put an end to all further controversy. The table containing Mr. Cricghton's fifteen cases, detailed from day to day, is peculiarly satisfactory, and though too long for publication in our Journal, ought to be carefully kept in the archives of the society. The author, however, in his very short analysis ("On the third day the punctures, &c.") has conveyed the substance. We think there was peculiar propriety in Mr. Cricghton's calling upon another gentleman to inoculate; and the success of Mr. Stewart, surgeon general, in producing the effect [local] in every subject, shews the matter to have been active and well applied. EDIT.

